# Tuning oxygen-containing functional groups of graphene for supercapacitors with high stability†

**DOI:** 10.1039/d2na00506a

**Published:** 2023-01-10

**Authors:** Shiqi Lin, Jie Tang, Kun Zhang, Youhu Chen, Runsheng Gao, Hang Yin, Lu-Chang Qin

**Affiliations:** a National Institute for Materials Science 1-2-1 Sengen Tsukuba Ibaraki 305-0047 Japan tang.jie@nims.go.jp; b University of Tsukuba 1-1-1 Tennodai Tsukuba Ibaraki 305-0006 Japan; c Department of Physics and Astronomy, University of North Carolina at Chapel Hill Chapel Hill NC 27599-3255 USA

## Abstract

To investigate the relationship between the oxygen-containing functional groups of graphene and the stability of supercapacitors, reduced graphene oxide (rGO) containing different oxygenic functional groups was prepared by varying the reduction time of GO using hydrazine as the reducing agent. TEM, XRD, Raman, and XPS characterizations revealed that, as the reduction time increased, the sp^2^ structure in the rGO sheet was restored and the obtained rGO had good crystallinity accompanied by removal of the oxygenic functional groups. The analysis of the content of the different functional groups also suggested that the reduction rate of the oxygenic functional group was C–O > C=O > O–C=O. The supercapacitive performance of rGO showed that the oxygenic functional groups contributed to some pseudocapacitance and resulted in a larger specific capacitance. At the same time, however, it is also accompanied by poorer rate performance and durability, which will be improved by removing the oxygenic functional groups by extending the reduction time. With an optimized reaction condition of a reduction time of 24 h, the obtained rGO exhibited excellent stability in floating tests at 3.0 V and 45 °C for 60 days. These findings pave the way for the development of high quality graphene materials for cost-effective and practical graphene supercapacitors.

## Abstract 2

This study aims to investigate the relationship between the residual functional groups on graphene sheets and the stability of graphene supercapacitors, thereby improving the performance of graphene supercapacitors.

## Graphical abstract

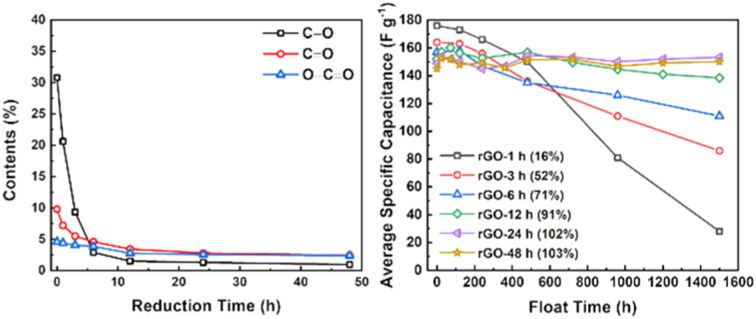

## 1. Introduction

With the rapid development of industrial applications, the vast consumption of oil has resulted in major environmental issues, such as greenhouse gas emissions, and has necessitated the development of alternative energy sources.^1–5^ Renewable energy sources, such as solar, wind and hydro energy, are considered ideal and promising energy sources to replace fossil fuels.^6–9^ However, due to the large fluctuations in the generation of electricity and the time lag between the production and use of electricity, there is an increasing demand for the storage of the electricity generated.^10,11^ Thus, it is important to develop efficient, high-performance, environmentally friendly, and low-cost energy storage devices and related energy storage materials. Among the various energy storage devices, supercapacitors (SCs) have attracted tremendous attention due to their rapid charging and discharging, high power density, and outstanding cycling stability.^12–18^ As for the electrodes, carbon materials have been one of the most widely studied and used materials in supercapacitors due to their abundant sources, low cost, good chemical stability, easy processing, and high specific surface area. Graphene, a two-dimensional carbon nanomaterial with a large specific surface area, high electrical conductivity, high mechanical strength, and good chemical stability, is considered as a most promising electrode material for supercapacitors.^19–21^ Various methods have been reported to synthesize graphene, including mechanical exfoliation, liquid-phase exfoliation, chemical vapor deposition, epitaxial growth, electrochemical deposition, microwave processing, and chemical reduction of graphene oxide (GO). Chemical reduction is the most widely used method due to its simplicity, low cost, and easy processability for high production.^19,20,22–32^ However, there are still some issues in the application of graphene materials for supercapacitors.

The current research on the application of graphene in supercapacitors mainly focuses on the preparation of graphene material with large capacitance by tuning the synthetic parameters.^33,34^ Although the stability of supercapacitors is a key factor for their practical applications, to the best of our knowledge, there were very few studies on the relationship between the reduction process of GO and the stability of graphene supercapacitors. The residual functional groups on the graphene sheet after chemical reduction will usually lead to degradation of the electrolyte. There are several oxygen-containing functional groups attached to the graphitic carbon plane in the structure of GO, including epoxide, hydroxyl, carbonyl, and carboxyl, making GO a hydrophilic substance.^35,36^ Furthermore, when GO is reduced to graphene, many of the hydrophilic groups on GO are removed, making the obtained graphene become hydrophobic, and this process will in turn hinder the re-stacking of graphene layers.

To address the abovementioned issues, this study aims to optimize the structure of the residual functional groups on graphene sheets by tuning the reduction parameters, thereby understanding the relationship between the residual functional groups and the stability of graphene supercapacitors, and improving the performance of graphene supercapacitors.

## 2. Experimental

### 2.1. Synthesis of graphene

Graphene was synthesized by chemical reduction of graphene oxide (GO) using hydrazine as the reducing agent.^34,37,38^ GO was first obtained from graphite by a modified Hummers' method.^39^ 5 g of natural graphite (Alfa), 3.75 g of NaNO_3_, and 310.5 g of H_2_SO_4_ were placed in a beaker and stirred for 30 min in an ice bath at 0 °C. Under vigorous agitation, 22.5 g of KMnO_4_ was then added slowly to the above solution and the temperature was kept at below 10 °C. The resultant mixture was then stirred for 2 days at room temperature, after which 1 L of H_2_SO_4_ aqueous solution (5%) was added drop by drop over a period of 1 h. After stirring for 2 h, 150 g of H_2_O_2_ (30%, Aldrich) was added to the mixture, making the color of the suspension change from brown to yellow. The GO suspension was allowed to settle down overnight and was then diluted by using 0.1 mol L^−1^ HCl, centrifuged 5 times, diluted with deionized water, and continuously centrifuged (30 000 rpm) until the pH value of the supernatant reached 7 to remove impurities.

The obtained GO solution was reduced to graphene by hydrazine using chemical reduction with different concentrations of GO for different reduction times, rinsed with ethanol, and finally dried in a vacuum at 60 °C.^40–42^ The chemical reactions of GO, with the concentration fixed at 2.0 g L^−1^, were carried out for different reduction times (1 h, 3 h, 6 h, 12 h, 24 h and 48 h) to explore the effect of reduction time on the structure and properties of the graphene synthesized. The concentration of hydrazine was 10 mL/1 g GO. The samples obtained with different reduction times were denoted as rGO-t-1, rGO-t-2, rGO-t-3, rGO-t-6, rGO-t-12, rGO-t-24, and rGO-t-48, respectively.

### 2.2. Microstructure characterization

Morphology of the synthesized graphene was characterized by both scanning electron microscopy (SEM, JSM-6500F/JSM-7001F, JEOL) and transmission electron microscopy (TEM, JEM-2100, JEOL). Atomic force microscopy (AFM) samples were prepared by spin-coating the rGO dispersion on a Si wafer. AFM measurements were conducted with a scanning probe microscope (JSPM-5200, JEOL) using the tapping mode. The structure was also examined by using powder X-ray diffraction (XRD, Rigaku SmartLab using Cu-Kα radiation with *λ* = 1.5418 Å) and Raman spectroscopy (Nanophoton Raman Plus, *λ* = 532 nm). The functional groups on GO and graphene were characterized with X-ray photoelectron spectroscopy (XPS, ULVAC-PHI Quantera SXM) and Fourier transform infrared spectroscopy (FTIR, Shimadzu, IRTracer-100). Elemental analyses were performed with energy-dispersive X-ray spectroscopy (EDS, JED-2300, JEOL). Nitrogen adsorption–desorption data (Quantachrome Autosorb iQ) were collected to calculate the specific surface area by the Brunauer–Emmett–Teller (BET) method and the distribution of pore sizes was obtained using density functional theory (DFT) calculations.

### 2.3. Electrochemical measurement

To evaluate the electrochemical performance of the synthesized graphene films, coin cells were assembled using a graphene film with a diameter of 15 mm as the electrodes, a layer of glass-fibre membrane as the separator, and two pieces of aluminium foil as the current collectors. 1-Ethyl-3-methylimidazolium tetrafluoroborate (EMI-BF_4_) was employed as the electrolyte in the coin cell. The mass of each electrode was about 3 mg. To avoid the influence of moisture in air, all operations were carried out in a glove box filled with Ar.

All electrochemical tests were carried out in a two-electrode system using an electrochemical workstation (Biologic VSP-300). Cyclic voltammetry (CV) measurements were conducted at different voltage scan rates ranging from 10 mV s^−1^ to 200 mV s^−1^. Galvanostatic charge–discharge curves (GCC) were obtained at current densities varying from 0.1 to 20 A g^−1^. The CV and GCC tests of supercapacitors in EMI-BF_4_ were performed in the range from 0 to 3.7 V. Electrochemical impedance spectroscopy (EIS) measurement was also performed over the frequency range from 10 kHz to 0.1 Hz. The coin cell was subjected to a floating test (voltage holding) at a voltage of 3.0 V and temperature of 45 °C in an oven. The specific capacitance was calculated from the GCC curves ranging from 0 to 3.7 V at a constant current density of 0.2 A g^−1^ after 1 day, 3 days, 5 days, 10 days, 15 days, 20 days, 30 days, 40 days, 50 days, and 60 days of floating. The cyclic performance measurements were conducted at a charge–discharge current density of 1 A g^−1^ using a Land battery test system.

Based on the results of the GCC measurements, the specific capacitance was calculated in accordance with the discharging time at each current density by using the following equation:^16,43^1
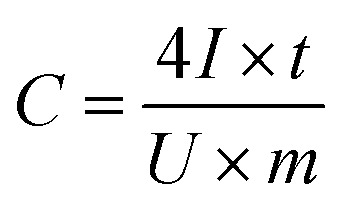
where *C* is the specific capacitance of the supercapacitor, *m* is the mass of two electrodes, *I*/*m* is the applied current density, *t* is the discharging time, and *U* is the potential window.

## 3. Results and discussion

### 3.1. Synthesis and characterization of rGO

**Fig. 1:**
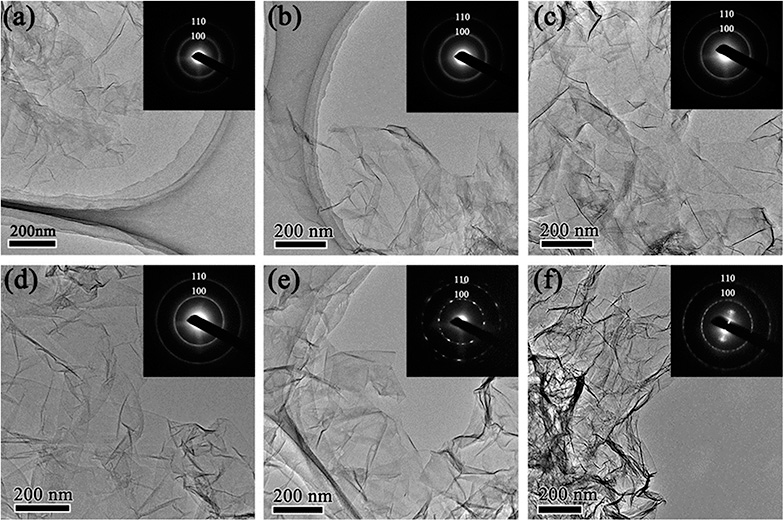
Low magnification TEM images of rGO with reduction time of (a) 1 h; (b) 3 h; (c) 6 h; (d) 12 h; (e) 24 h; (f) 48 h. Insets are the corresponding selected-area electron diffraction (SAED) patterns. Diffraction rings in the SAED patterns indicate the Bragg reflections corresponding to the (100) and (110) planes of graphene.

The morphology of rGO synthesized with various reduction times was studied by transmission electron microscopy (TEM). Fig. 1 shows low magnification TEM images of rGO with reduction times of 1 h (rGO-t-1), 3 h (rGO-t-3), 6 h (rGO-t-6), 12 h (rGO-t-12), 24 h (rGO-t-24), and 48 h (rGO-t-48). These rGOs exhibit a folded structure, with wrinkles on its two-dimensional plane, and tend to fall on each other. Besides, with the increase of reduction time, the rGO sheets tend to aggregate together. Selected-area electron diffraction (SAED) was also carried out to evaluate the crystallinity of graphene, which was helpful to understand the removal of oxygen-containing functional groups. The disordered distribution of oxygenic functional groups on the surface of graphene caused the diffraction rings to be blurred and broad.^44^ The effective removal of oxygenic functional groups would restore the six-fold symmetry of graphene. When the reduction time is below 12 h, the diffraction rings corresponding to the (100) and (110) planes of graphene are blurred and broad, suggesting a heavy presence of residual oxygenic functional groups. When the reduction time is more than 12 h, the diffraction rings corresponding to the (100) and (110) planes of graphene can be observed clearly, indicating the effective removal of oxygenic functional groups. Moreover, the diffraction spots became bright and sharp from graphene after reduction for 24 h, which indicated a sufficient removal of oxygenic functional groups and the restoration of the sp^2^ bonding of graphitic carbon.

**Fig. 2:**
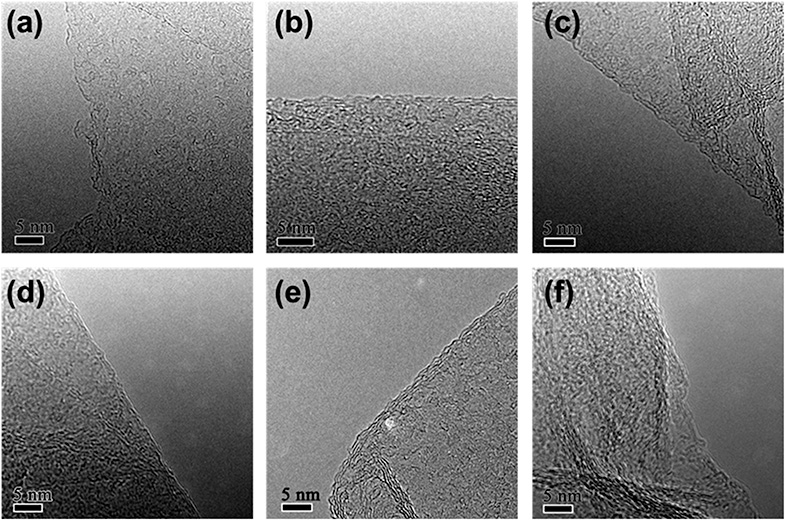
High-resolution TEM images of rGO with reduction time of (a) 1 h; (b) 3 h; (c) 6 h; (d) 12 h; (e) 24 h; (f) 48 h.

High-resolution TEM (HRTEM) images of folded graphene edges were further used to reveal the number of stacked layers of graphene as illustrated in Fig. 2. Multi-layered graphene is a result of re-stacking of single-layered graphene *via* van der Waals interactions, which is difficult to redisperse even with extended sonication. The re-stacking of graphene will lead to a decrease in the specific surface area of materials. Fig. 2 reveals that the number of stacked layers increases with increasing reduction time, suggesting simultaneous increase of the restacking of graphene. Generally, when the reduction time is less than 24 h, graphene usually consists of few-layered nanosheets (1–5 layers), while they would have more multi-layered graphene nanosheets (3–10 layers) in the case of more than 24 h. Furthermore, the thickness of the obtained rGO sheets was also analysed using AFM. Fig. S1† shows a typical AFM image of rGO-t-3, showing rGO sheets of micron size overlapped with several other rGO sheets, similar to those observed with TEM and SEM. The partial stacking is resulted from the evaporation of the solvent in the sample preparation process. The rGO-t-3 sheet has a thickness ranging from 0.79 nm to 1.78 nm, corresponding to 2–5 graphene layers. On the other hand, rGO-t-24 has a thickness ranging from 2.13 nm to 3.87 nm (Fig. S2†), corresponding to 6–11 graphene layers. A structural study of graphene reveals that a longer reduction time is helpful for the removal of oxygenic functional groups but will promote the re-stacking of graphene. A reduction time of 24 h is an optimum condition to sufficiently remove the oxygenic functional groups and reduce the re-stacking of graphene (3–8 layers).

**Fig. 3:**
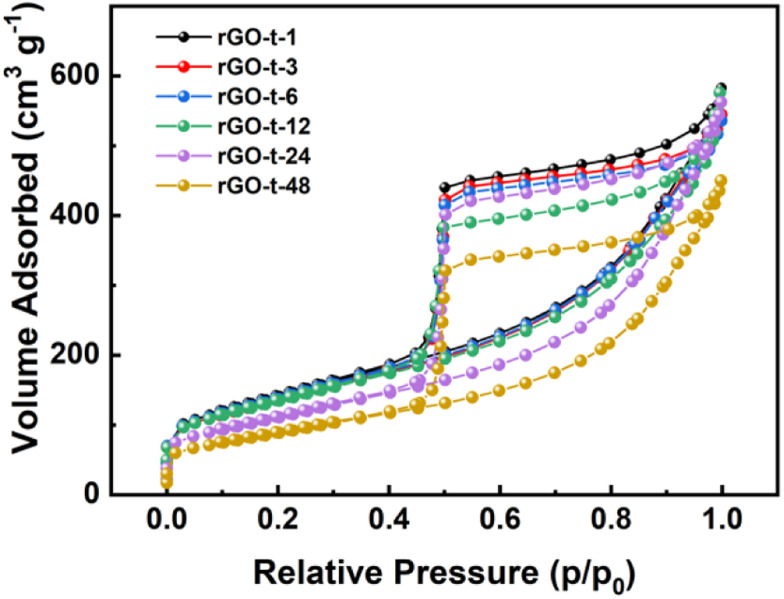
Nitrogen adsorption/desorption isotherms of rGO synthesized with different reduction times (1 h, 3 h, 6 h, 12 h, 24 h, and 48 h).

To characterize the specific surface area of the rGO synthesized with different reduction times, nitrogen adsorption/desorption isotherms were obtained, and the results are displayed in Fig. 3. In accordance with the IUPAC classification, all the curves exhibit a typical type IV isotherm, indicating the presence of micropores and mesopores. And the specific surface areas of the rGOs are 506 m^2^ g^−1^ (rGO-t-1), 487 m^2^ g^−1^ (rGO-t-3), 491 m^2^ g^−1^ (rGO-t-6), 481 m^2^ g^−1^ (rGO-t-12), 440 m^2^ g^−1^ (rGO-t-24), and 404 m^2^ g^−1^ (rGO-t-48), corresponding to reduction time of 1 h, 3 h, 6 h, 12 h, 24 h, and 48 h, respectively, as illustrated in Fig. 3. The reason for the decrease in the specific surface area of rGO with increasing reduction time is the re-stacking of rGO sheets, as also confirmed by the TEM observations.^45,46^

**Fig. 4:**
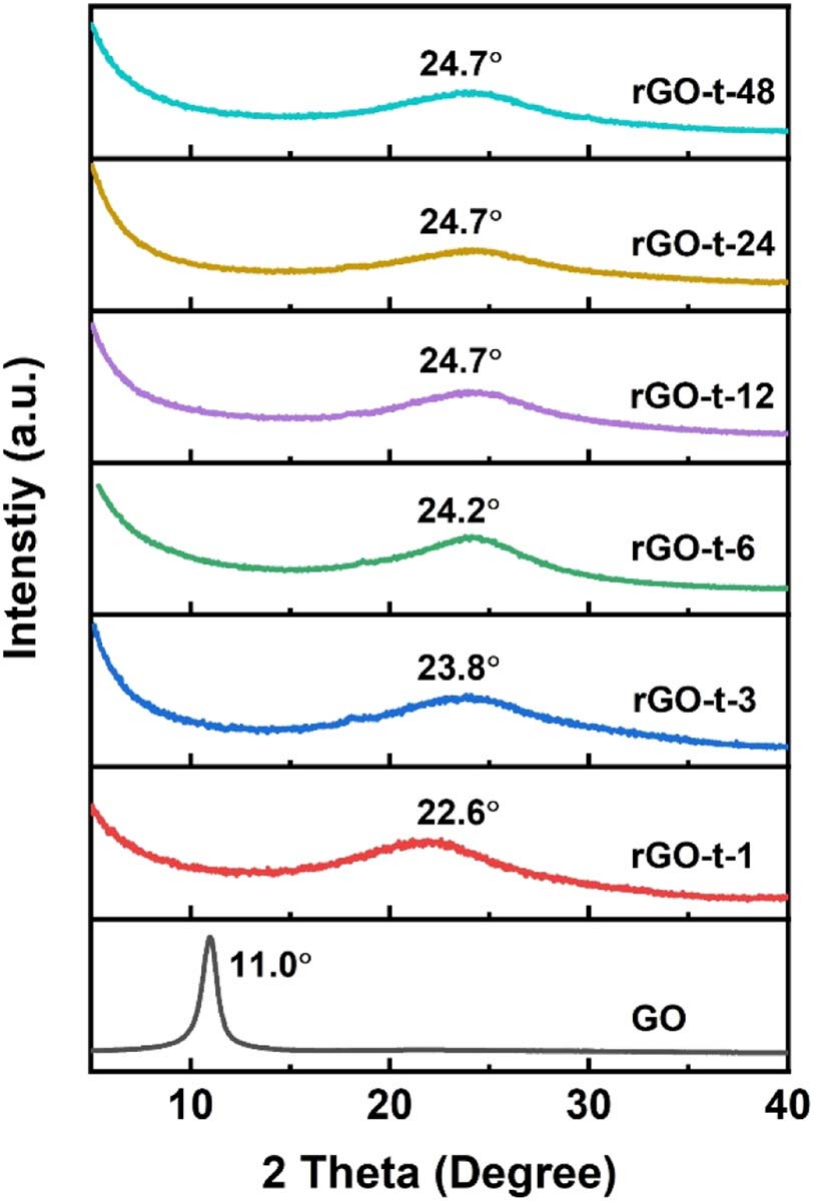
XRD patterns of GO and rGO prepared with various reduction times.

To further characterize the evolution of the microstructure of GO during reduction, the XRD patterns of rGOs synthesized with various reduction times were collected and analysed. As shown in Fig. 4, GO exhibited a sharp diffraction peak at 2*θ* = 11.0° (corresponding to a *d*-spacing of 0.80 nm). Compared with the typical (002) diffraction peak of graphite (2*θ* = 26.6°, corresponding to a *d*-spacing of 0.34 nm) as shown in Fig. S3,† the increase in the *d*-spacing of GO is due to the oxygenic functional groups (epoxy, hydroxyl, carboxyl, and carbonyl groups) in the basal plane of GO. After 1 h reduction by hydrazine, the sharp diffraction peak of GO at 2*θ* = 11.1° disappeared, and a new broad diffraction peak of rGO appeared at 2*θ* = 22.6° (corresponding to a *d*-spacing of 0.39 nm). With the increase of reaction time, the diffraction peak of the obtained rGO shifted to a higher angle and stayed almost constant (at 2*θ* = 24.7°, corresponding to a *d*-spacing of 0.36 nm) after the reaction time reached 12 h. This result suggests that these oxygenic functional groups had an important effect on the interlayer spacing of rGO. With the reduction of GO, most of the oxygenic functional groups were removed, and the interlayer spacing of the resulting rGO gradually decreased. In addition, the removal of functional groups also led to the re-stacking of graphene sheets due to van der Waals forces to create a thicker layer, which is consistent with the features observed in the TEM images.^47^

**Fig. 5:**
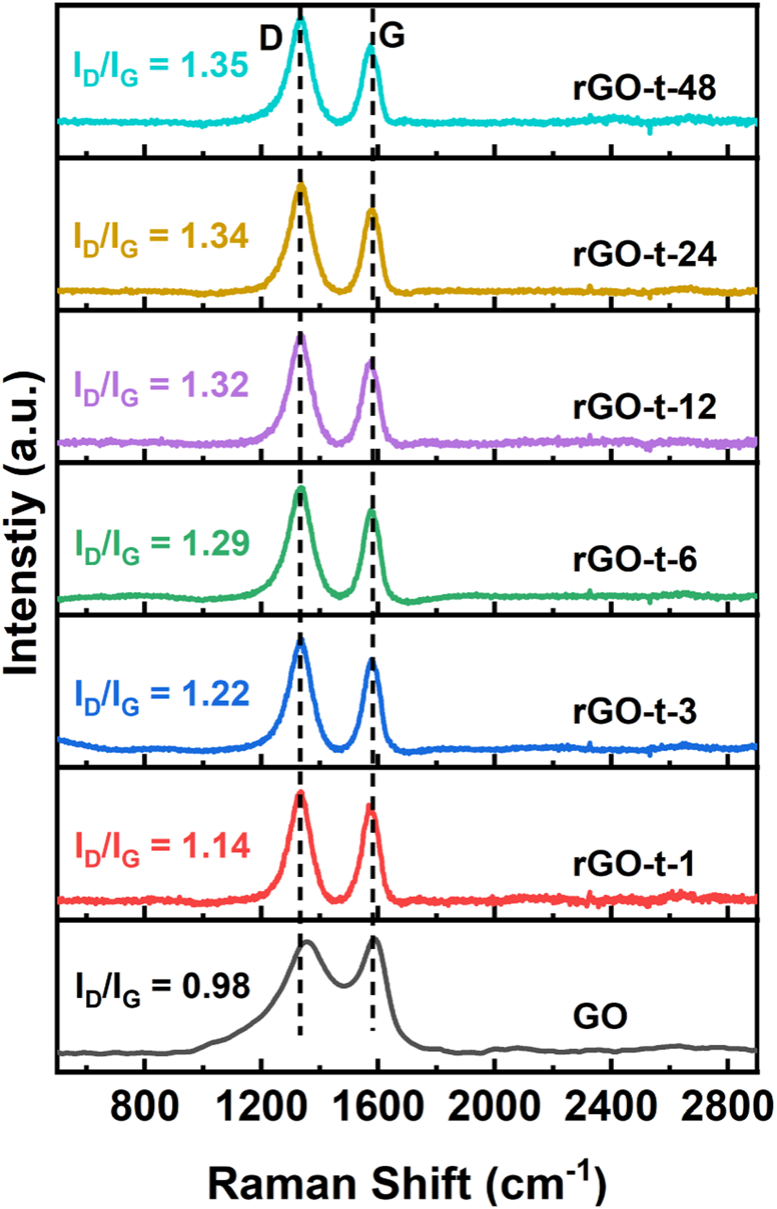
Raman spectra of GO and rGO prepared with various reduction times.

Raman spectroscopy is a widely used technique to characterize the structural characteristics and properties of graphene materials. The G peak at ∼1580 cm^−1^ represents the in-plane stretching vibrations of the sp^2^ bonded carbon, and the D peak at ∼1350 cm^−1^ represents the defective and disordered structures in the hexagonal lattice of graphene. The intensity ratio of the D peak and G peak (*I*_D_/*I*_G_) is often useful to evaluate the degree of defective structure of carbon materials. Fig. 5 shows the Raman spectra of GO and rGO synthesized with various reduction times. For GO, the G peak is located at 1687 cm^−l^, while the G peak of rGO moved to 1575 cm^−1^, which is attributed to a graphitic “self-healing” with the removal of oxygenic functional groups and restoration of the hexagonal structure of graphene.^48^ The *I*_D_/*I*_G_ ratio for GO is 0.98 and it increased to 1.14 for rGO-t-1, indicating the introduction of more lattice defects accompanied by removal of the oxygenic functional groups in the reduction process. This is because, although the reduction process restores the conjugated sp^2^ carbon structures, the accompanying removal of carbon atoms bonded to the oxygenic functional groups would leave some vacancies and the defects would eventually lead to an increase in the *I*_D_/*I*_G_ value. As shown in Fig. 5, the value of *I*_D_/*I*_G_ increased from 1.14 to 1.34 when the reduction time increased from 1 h to 24 h and remained almost unchanged with further prolongation of the reduction time to 48 h, indicating that the degree of reduction reached its limitation.

**Fig. 6:**
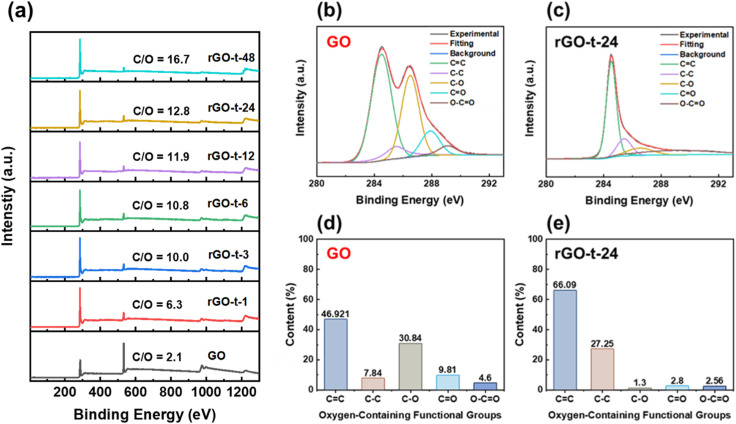
(a) XPS survey spectra of GO and rGOs synthesized with various reduction times. C 1s XPS spectra of (b) GO and (c) rGO-t-24. Percentage of individual oxygenic functional groups of (d) GO and (e) rGO-t-24.

XPS measurements were also employed to monitor the reduction behaviour of rGO. The XPS survey spectra of GO and rGOs with various reduction times are shown in Fig. 6a. The GO presents a strong O 1s peak with an oxygen concentration of 32.2%, corresponding to an atomic C/O ratio of 2.1. After reduction, the C/O ratio increased from 2.1 for GO to 6.3 (rGO-t-1), 10.0 (rGO-t-3), 10.8 (rGO-t-6), 11.9 (rGO-t-12), 12.8 (rGO-t-24), and 16.7 (rGO-t-48) for rGO with a reduction time of 1 h, 3 h, 6 h, 12 h, 24 h, and 48 h, respectively. Specifially, the C/O ratio (rGO-t-48) is in good agreement with the theoretical simulation value of 16.0.^49^ These results suggest that the oxygenic functional groups were effectively removed from the carbon planes during the reduction process. Furthermore, an EDS elemental analysis of the obtained rGO was also carried out (Fig. S4†). The C/O ratios are 7.2 (rGO-t-1), 9.8 (rGO-t-3), 10.8 (rGO-t-6), 11.7 (rGO-t-12), 12.9 (rGO-t-24), and 17.1 (rGO-t-48) for rGO with a reduction time of 1 h, 3 h, 6 h, 12 h, 24 h, and 48 h, respectively. These EDS measurements are consistent with the XPS analyses.

To obtain a quantitative picture of the evolution of the oxygenic functional groups during the reduction process, the C 1s XPS spectra of GO and rGOs were deconvoluted into five peaks at binding energies of 284.5 eV, 285.5 eV, 286.5 eV, 287.9 eV, and 289.1 eV, corresponding to the sp^2^-hybridized carbon (C=C), sp^3^-hybridized carbon (C–C), hydroxyl and epoxy groups (C–O), carbonyl groups (C=O), and carboxyl groups (O–C=O), respectively.^33,46^ To monitor the evolution of individual functional groups during the reduction process, the contents of each functional group were estimated by the percentage of its area relative to the whole area of the C 1s peak. Fig. 6b and c show the C 1s XPS spectra of GO and rGO-t-24, and the contents of the individual functional groups are plotted in histograms shown in Fig. 6d and e. According to Fig. 6d and e, it is clear that C–O is the major component in GO. And the histograms show the value of the peak area of each type of the oxygenic functional group. Compared with GO, after chemical reduction by hydrazine, the intensity of the C–O group was significantly decreased, while the C=C bonding and C–C bonding increased correspondingly. The relative proportion of the C=C bonds increased from 46.92% (for GO) to 66.09% (for rGO-t-24), and the relative proportion of the C–C bonds increased from 7.84% (for GO) to 27.25% (for rGO-t-24), while the relative proportion of the C–O group decreased significantly from 30.84% (for GO) to 1.30% (for rGO-t-24), which implies that GO was reduced to graphene effectively by hydrazine.

**Fig. 7:**
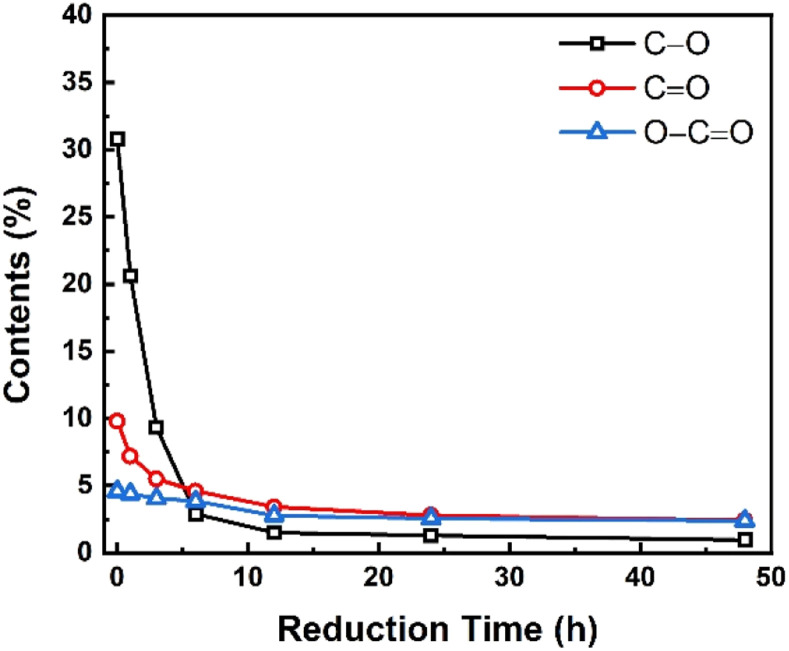
Changes in various oxygenic functional groups in graphene synthesized with different reduction times.

Fig. 7 depicts the evolution of hydroxyl and epoxy groups (C–O), carbonyl groups (C=O), and carboxyl groups (O–C=O) during the reduction process. As shown in Fig. 7, the relative contents of all the three types of functional groups were decreased with the increase in reduction time, suggesting the removal of oxygenic functional groups and restoration of the conjugated sp^2^ graphitic structures. From the changing trend of each oxygenic functional group with the reduction time, each type of functional group changed significantly in the first 5 h. After that, the trend of change slowed down and it remained almost unchanged after 12 h, implying that the chemical reduction mainly occurred in the initial stage. And regarding the reduction rate of the various functional groups, the hydroxyl and epoxy groups (C–O) are the fastest ones, followed by the carbonyl groups (C=O), and the slowest ones were the carboxyl groups (O–C=O). It was also found that the contents of C–O, C=O, and O–C=O groups in GO were 30.8%, 9.8%, and 4.6%, respectively, but after 48 h reduction, these three types of functional groups in rGO-t-48 were decreased to 1.0%, 2.4%, and 2.4%, respectively. It means that 96.8% C–O, 75.5% C=O, and 47.8% O–C=O functional groups were removed after 48 h reduction, indicating that the C=O and O–C=O groups were more stable and less likely to react with hydrazine. The C–O groups are expected to react first in the presence of hydrazine since they are single bonded groups with a bonding energy of 358 kJ mol^−1^,^50^ while the C=O groups are double bonded functional groups with a higher bonding energy of 745 kJ mol^−1^ and need more energy to break them apart.^50^

### 3.2. Electrochemical measurements

**Fig. 8:**
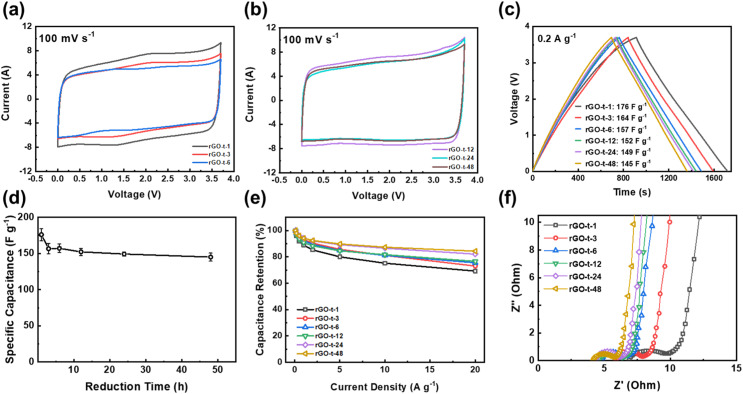
CV curves at voltage sweep rate of 100 mV s^−1^ of graphene synthesized with reduction time of (a) 1 h, 3 h, and 6 h; (b) 12 h, 24 h, and 48 h; (c) charge–discharge curves, (d) specific capacitance, (e) rate performance, and (f) Nyquist plots of supercapacitors with rGOs synthesized with different reduction times.

As mentioned in Introduction, the aim of this work was to study the relationship between the structure of rGO and its supercapacitor performance and stability. To examine the supercapacitor performance and stability of the rGOs synthesized with various reduction times, symmetric two-electrode cells were assembled using EMI-BF_4_, an ionic liquid with a large electrochemical window up to 3.7 V, as the electrolyte. Fig. 8a and b display the CV curves of rGO based supercapacitors at a scanning rate of 100 mV s^−1^. As shown in Fig. 8a and b, all CV curves exhibited a near-rectangular shape, revealing characteristics of an electrochemical double-layer capacitor (EDLC). Comparing the CV curves of rGOs obtained at different reduction times, it is clear that the CV curves of rGO-t-1 and rGO-t-3 exhibited a pair of redox peaks (Fig. 8a). And with the increase of the reduction time, this pair of redox peaks gradually weakened and completely disappeared in the CV curves of rGO obtained with a reduction time greater than 12 h (Fig. 8b). This pair of redox peaks is attributed to the electrochemical reactions of oxygenic functional groups on the surface of rGO. With the increase in reduction time, the oxygenic functional groups on the surface of the obtained rGO decreased (as shown in Fig. 8b), and the peaks in CV became weakened accordingly. It is also worth noting that these electrochemical reactions also contributed a small amount of pseudocapacitance, as we can see that the CV curves of rGO-t-1 and rGO-t-3 have larger areas compared to other CV curves.

As shown in Fig. 8c, their charge–discharge curves at current density of 0.2 A g^−1^ have triangular shapes. Consistent with the results obtained in the CV curves, the charge–discharge curve of rGO-t-1 exhibited a slight deviation from the linear slope of an ideal triangular shape, indicating a pseudocapacitive behaviour due to the presence of a large number of oxygen-containing functional groups with a C/O ratio of 6.3. With an increase in the reduction time, the charge–discharge curves of the obtained rGO returned to the ideal isosceles triangular shape, suggesting an excellent capacitive behaviour with further removal of the oxygenic functional groups. The specific capacitance calculated from the charge–discharge curves of rGO with different reduction times is plotted in Fig. 8d. It can be seen that the capacitance of rGO decreased from 176 F g^−1^ (rGO-t-1) to 157 F g^−1^ (rGO-t-6) when the reduction time was increased from 1 h to 6 h, due to the disappearance of the contributions of pseudocapacitance accompanied by the removal of oxygenic functional groups. Moreover, as the reduction time was further increased to 48 h, the capacitance of rGO was maintained at about 150 F g^−1^ without any obvious decrease. When the current density was increased from 0.2 to 20 A g^−1^, the specific capacitance of rGO-t-1 exhibited a large decrease, and the capacitance retention was only 69% as shown in Fig. 8e. However, the specific capacitance of rGO-t-48 showed only a slight decrease with a higher capacitance retention rate of 85%, which proved that the rGO sample obtained with a longer reduction time had an excellent rate capability.

The rate performance was also verified by the EIS measurements. Fig. 8f shows the Nyquist plots for the graphene supercapacitors that showed a semicircle in the high-frequency region and a 45° straight line in the low-frequency region and the intermediate-frequency region. As shown in Fig. 8f, the equivalent series resistance (*R*_s_) of rGO is 6.9 Ω, 5.9 Ω, 4.8 Ω, 4.5 Ω, 4.3 Ω and 4.3 Ω for rGO-t-1, rGO-t-3, rGO-t-6, rGO-t-12, rGO-t-24, and rGO-t-48, respectively, indicating an increase of the conductivity of the rGO film with the increase of reduction time. The semicircle in the high frequency region represents a charge transfer resistance (*R*_ct_) and the diameter of the semicircle is related to the diffusion of charge carriers at the electrode/electrolyte interface. The continuous decrease in the diameter of the semicircle from rGO-t-1 to rGO-t-48 reveals the decrease of *R*_ct_ with increasing reduction time of rGO. These results indicate that rGO obtained with a longer reduction time, accompanied by the removal of more oxygenic functional groups and restoration of the conjugated sp^2^ carbon structures as discussed above, on the one hand, facilitated electrolyte ions to be reversibly physiosorbed/desorbed on the surface of graphene, while on the other hand, improved the conductivity of rGO and allowed fast electron transfers. All these factors contributed significantly to the excellent rate performance of the graphene electrode obtained with a longer reduction time. Furthermore, the straight line in the low frequency region can explain the capacitive characteristics of the electrode material. The relatively smaller slopes in rGO-t-1 and rGO-t-3 indicate a deviation from the ideal capacitive behaviour, while the slopes for rGO with increasing reduction time approached being vertical, suggesting a return to the ideal capacitive behaviour. These features are in agreement with their CV and charge–discharge curves.

**Fig. 9:**
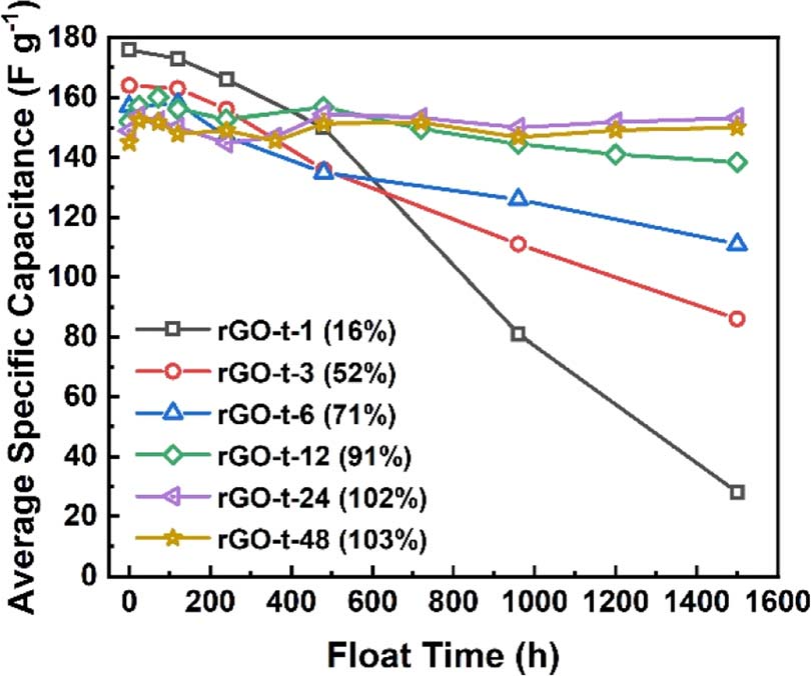
The average specific capacitance of graphene synthesized with different reduction times after the floating test (voltage holding) at voltage of 3.0 V and temperature of 45 °C in an oven for 1 day, 3 days, 5 days, 10 days, 15 days, 20 days, 30 days, 40 days, 50 days, and 60 days.

To characterize the stability of the graphene electrode, supercapacitor cells were tested by a very hard constant-voltage holding method with an applied voltage of 3.0 V at an atmospheric temperature of 45 °C for a period of 1500 h. As shown in Fig. 9, the capacitance of rGO-t-1 exhibited a near-linear decrease during the whole test process, and its capacitance retention was only 16% after 1500 h, while the other rGOs exhibited no decrease in capacitance within the first 120 h, and all maintained a good stability. Furthermore, a slight increase in capacitance was also observed. It is attributed to the electrochemical activation effect which allowed ions to intercalate into the stacked rGO sheets and created more surfaces for ion storage. However, rGO-t-3, rGO-t-6, and rGO-t-12 also showed different degrees of capacitance decrease as the test progressed, and their capacitance retention rates after 1500 h were 52%, 71%, and 91%, respectively. In contrast, rGO-t-24 and rGO-t-48 retained their initial capacitance after 1500 h, demonstrating excellent stabilities in the hard accelerated stability test at high temperature and high voltage. In addition, cyclic tests of the supercapacitors with rGO electrodes at a current density of 1 A g^−1^ were also carried out at room temperature. As shown in Fig. S5,† the retention rates of specific capacitance after 10 000 cycles were 70%, 81%, 94%, 97%, 97%, and 99% for rGO-t-1, rGO-t-3, rGO-t-6, rGO-t-12, rGO-t-24, and rGO-t-48, respectively. These results once again confirmed the excellent stability of rGO-t-24 and rGO-t-48 electrodes.

## 4. Conclusions

In this study, we optimized the structure of residual functional groups on graphene sheets in the preparation of GO and established an effective process by tuning the operation parameters to reduce GO. With a comprehensive consideration of the synthesis parameters for graphene production and the performance of supercapacitors, we have developed a mechanism accounting for the structural and electrochemical properties. With an increase in reduction time, more oxygenic functional groups were removed. The reduction rate follows C–O > C=O > O–C=O. The performance of the graphene supercapacitor is largely determined by the carboxyl functional groups that remained on the basal planes of graphene. In the process of GO reduction, the reducing agent needs a longer time to reach these oxygenic functional groups to react with them. Combining the functional groups, the structure of graphene, and the results of durability tests, it indicates that the deterioration of graphene supercapacitors is a result of the redox reactions of the acidic carboxyl functional groups on the graphene sheets. It has guiding significance for the production of high-performance graphene for supercapacitor applications.

## Author contributions

Shiqi Lin: conceptualization, investigation, formal analysis, visualization, writing – original draft. Jie Tang: conceptualization, formal analysis, writing – review and editing, supervision, project administration, funding acquisition. Kun Zhang: visualization, validation, writing – review and editing. Youhu Chen: visualization. Runsheng Gao: visualization. Hang Yin: visualization. Lu-Chang Qin: formal analysis, visualization, writing – review and editing.

## Conflicts of interest

There are no conflicts of interest to declare.

## Supplementary Material

NA-005-D2NA00506A-s001

## References

[cit1] Raza W., Ali F., Raza N., Luo Y., Kim K., Yang J. (2018). Nano Energy.

[cit2] Béguin F., Presser V., Balducci A., Frackowiak E. (2014). Adv. Mater..

[cit3] Shao Y., El-kady M. F., Sun J., Li Y., Zhang Q., Zhu M., Wang H., Dunn B., Kaner R. B. (2018). Chem. Pap..

[cit4] Lashof D. A., Ahujah D. R. (1990). Nature.

[cit5] Chu S., Majumdar A. (2012). Nature.

[cit6] Hagfeldt A., Boschloo G., Sun L., Kloo L., Pettersson H. (2010). Chem. Rev..

[cit7] Dillon A. C. (2010). Chem. Rev..

[cit8] Christopher K., Dimitrios R. (2012). Energy Environ. Sci..

[cit9] Cook T. R., Dogutan D. K., Reece S. Y., Surendranath Y., Teets T. S., Nocera D. G. (2010). Chem. Rev..

[cit10] Article R. (2013). Chem. Soc. Rev..

[cit11] Miller J. R., Simon P. (2008). Science.

[cit12] Simon P., Gogotsi Y. (2008). Nat. Mater..

[cit13] Zhang L., Zhao X. S. (2009). Chem. Soc. Rev..

[cit14] Wang G., Zhang L., Zhang J. (2012). Chem. Soc. Rev..

[cit15] Zhai Y., Dou Y., Zhao D., Fulvio P. F., Mayes R. T., Dai S. (2011). Adv. Mater..

[cit16] Béguin F., Presser V., Balducci A., Frackowiak E. (2014). Adv. Mater..

[cit17] Joanni E., Kumar R., Fernandes W. P., Savu R., Matsuda A. (2022). Nanoscale.

[cit18] Sahoo S., Kumar R., Joanni E., Singh R. K., Shim J. J. (2022). J. Mater. Chem. A.

[cit19] Zhu Y., Murali S., Cai W., Li X., Suk J. W., Potts J. R., Ruoff R. S. (2010). Adv. Mater..

[cit20] Agarwal V., Zetterlund P. B. (2021). Chem. Eng. J..

[cit21] Pei S., Cheng H. M. (2012). Carbon.

[cit22] Kumar R., Sahoo S., Tan W. K., Kawamura G., Matsuda A., Kar K. K. (2021). J. Energy Storage.

[cit23] Kumar R., Sahoo S., Joanni E., Singh R. K. (2022). J. Energy Chem..

[cit24] Devi N., Sahoo S., Kumar R., Singh R. K. (2021). Nanoscale.

[cit25] Adetayo A., Runsewe D. (2019). Open J. Compos. Mater..

[cit26] Bonaccorso F., Lombardo A., Hasan T., Sun Z., Colombo L., Ferrari A. C. (2012). Mater. Today.

[cit27] Hernandez Y., Nicolosi V., Lotya M., Blighe F. M., Sun Z., De S., McGovern I. T., Holland B., Byrne M., Gun'ko Y. K., Boland J. J., Niraj P., Duesberg G., Krishnamurthy S., Goodhue R., Hutchison J., Scardaci V., Ferrari A. C., Coleman J. N. (2008). Nat. Nanotechnol..

[cit28] Yang W., Chen G., Shi Z., Liu C. C., Zhang L., Xie G., Cheng M., Wang D., Yang R., Shi D., Watanabe K., Taniguchi T., Yao Y., Zhang Y., Zhang G. (2013). Nat. Mater..

[cit29] Murdock A. T., Koos A., Ben Britton T., Houben L., Batten T., Zhang T., Wilkinson A. J., Dunin-Borkowski R. E., Lekka C. E., Grobert N. (2013). ACS Nano.

[cit30] Yi M., Shen Z. (2015). J. Mater. Chem. A.

[cit31] Youssry S. M., El-Hallag I. S., Kumar R., Kawamura G., Tan W. K., Matsuda A., El-Nahass M. N. (2022). J. Energy Storage.

[cit32] Kumar R., Youssry S. M., Soe H. M., Abdel-Galeil M. M., Kawamura G., Matsuda A. (2020). J. Energy Storage.

[cit33] Ren P. G., Yan D. X., Ji X., Chen T., Li Z. M. (2010). Nanotechnology.

[cit34] Park S., An J., Potts J. R., Velamakanni A., Murali S., Ruoff R. S. (2011). Carbon.

[cit35] Chandu B. (2021). Caribb. J. Sci. Technol..

[cit36] Chua C. K., Pumera M. (2014). Chem. Soc. Rev..

[cit37] Cheng Q., Tang J., Ma J., Zhang H., Shinya N., Qin L.-C. (2011). Carbon.

[cit38] Cheng Q., Tang J., Ma J., Zhang H., Shinya N., Qin L.-C. (2011). Phys. Chem. Chem. Phys..

[cit39] Hummers W. S., Offeman R. E. (1958). J. Am. Chem. Soc..

[cit40] Li J., Tang J., Yuan J., Zhang K., Sun Y., Zhang H., Qin L.-C. (2017). Electrochim. Acta.

[cit41] Li J., Tang J., Yuan J., Zhang K., Shao Q., Sun Y., Qin L.-C. (2016). Electrochim. Acta.

[cit42] Sun Y., Tang J., Zhang K., Yuan J., Li J., Zhu D. M., Ozawa K., Qin L.-C. (2017). Nanoscale.

[cit43] Nithya V. D. (2021). J. Energy Storage.

[cit44] Chen Y., Niu Y., Tian T., Zhang J., Wang Y., Li Y., Qin L.-C. (2017). Chem. Phys. Lett..

[cit45] Fan L. Z., Liu J. L., Ud-Din R., Yan X., Qu X. (2012). Carbon.

[cit46] Kuang B., Song W., Ning M., Li J., Zhao Z., Guo D., Cao M., Jin H. (2018). Carbon.

[cit47] Li D., Müller M. B., Gilje S., Kaner R. B., Wallace G. G. (2008). Nat. Nanotechnol..

[cit48] Stoller M. D., Park S., Zhu Y., An J., Ruoff R. S. (2008). Nano Lett..

[cit49] Boukhvalov D. W., Katsnelson M. I. (2008). J. Am. Chem. Soc..

[cit50] Shams M., Guiney L. M., Huang L., Ramesh M., Yang X., Hersam M. C., Chowdhury I. (2019). Environ. Sci.: Nano.

