# Supplementary material for: Tuning oxygen-containing functional groups of graphene for supercapacitors with high stability
Source: Nanoscale Adv. 2023 Jan 10;5(4):1163–71. doi: 10.1039/d2na00506a (PMC9926907; doi:10.1039/d2na00506a)
Supplement: NA-005-D2NA00506A-s001 [file NA-005-D2NA00506A-s001.pdf]

Electronic Supplementary Material (ESI) for Nanoscale Advances.
This journal is © The Royal Society of Chemistry 2022

Supplementary Information

Tuning Oxygen-Containing Functional Groups of Graphene for Supercapacitors with High Stability

Shiqi Lin [a,b], Jie Tang [a,b,*], Kun Zhang [a], Youhu Chen [a], Runsheng Gao [a], Hang Yin [a], Lu-Chang Qin[c]

[a] *National Institute for Materials Science, 1-2-1 Sengen, Tsukuba, Ibaraki 305-0047, Japan*

[b] *University of Tsukuba, 1-1-1 Tennodai, Tsukuba, Ibaraki 305-0006, Japan*

[c] *Department of Physics and Astronomy, University of North Carolina at Chapel Hill, Chapel Hill, NC 27599-3255, USA*

Corresponding author: Jie Tang, tang.jie@nims.go.jp

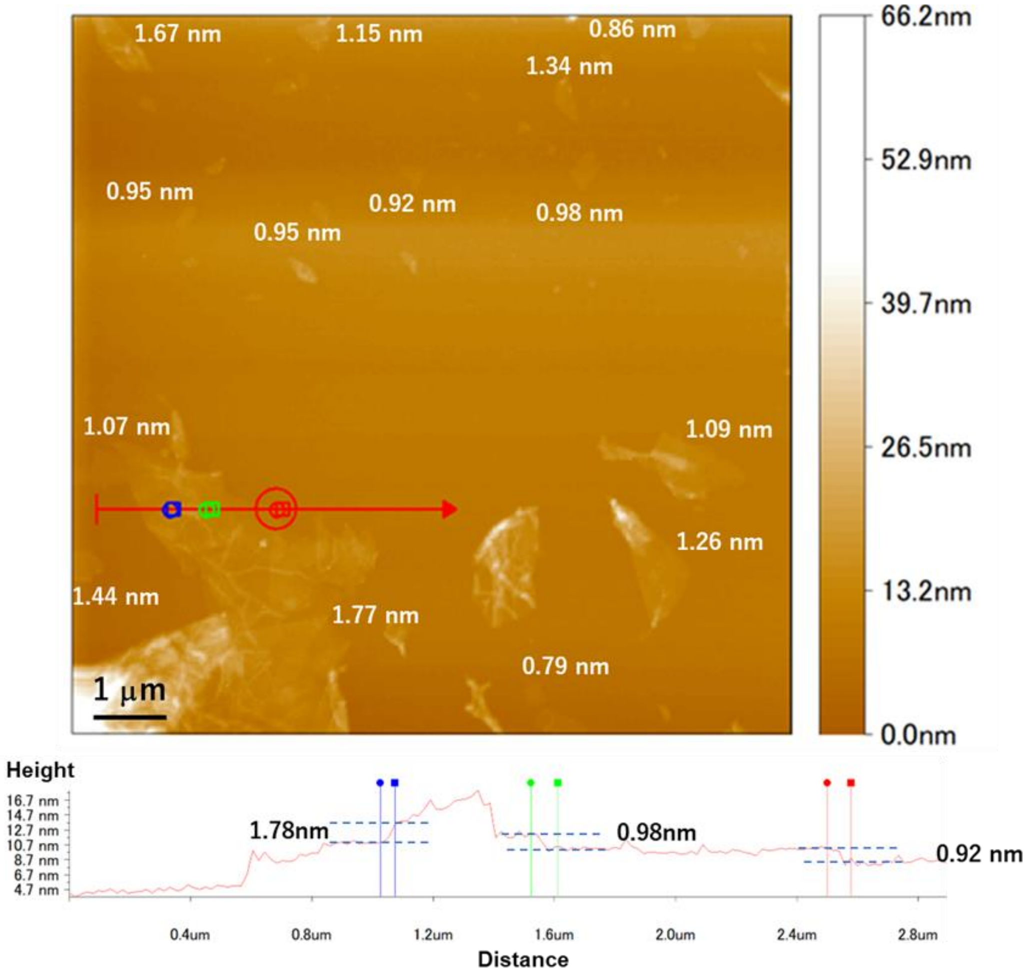

Fig. S1. AFM image (taken in tapping mode) of rGO-t-3 spin-coated onto Si wafer.

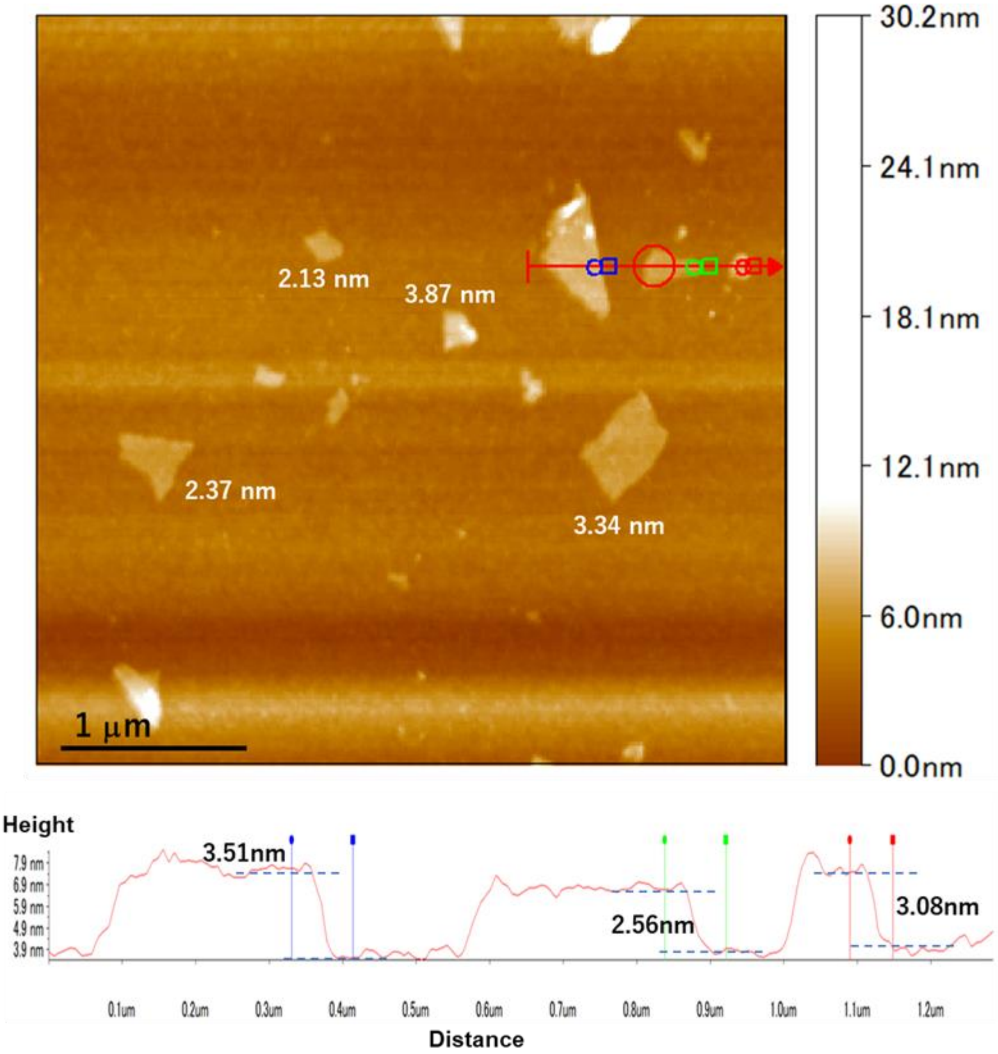

Fig. S2. AFM image (taken in tapping mode) of rGO-t-24 spin-coated onto Si wafer.

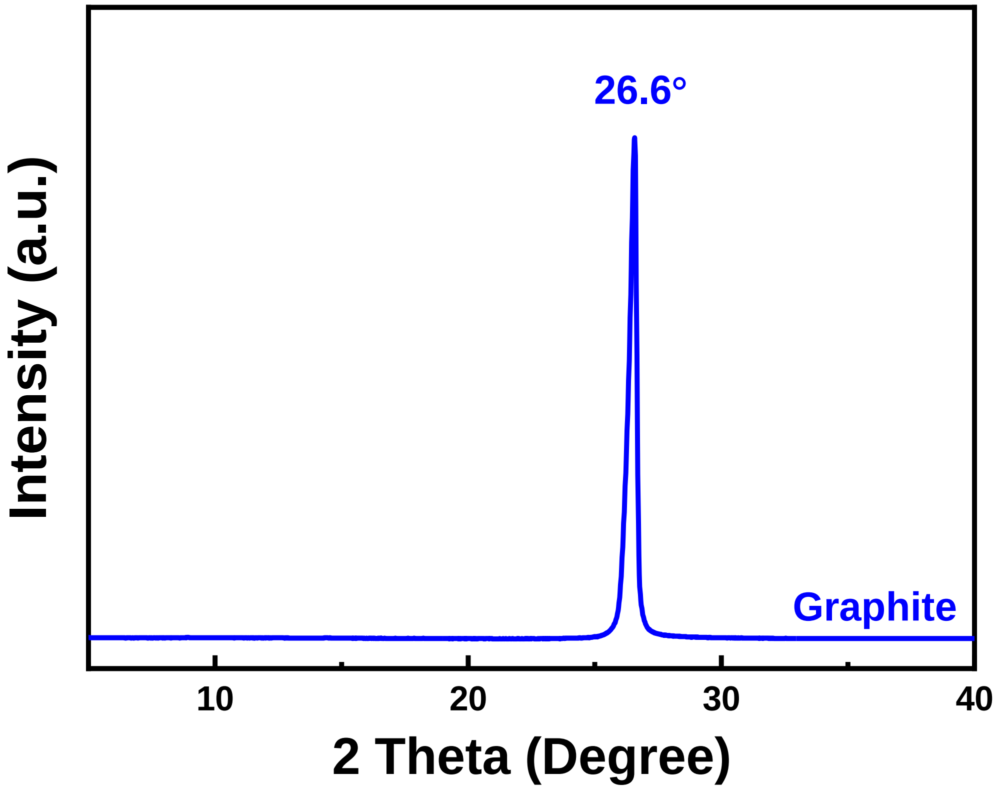

Fig. S3. XRD pattern of graphite powders.

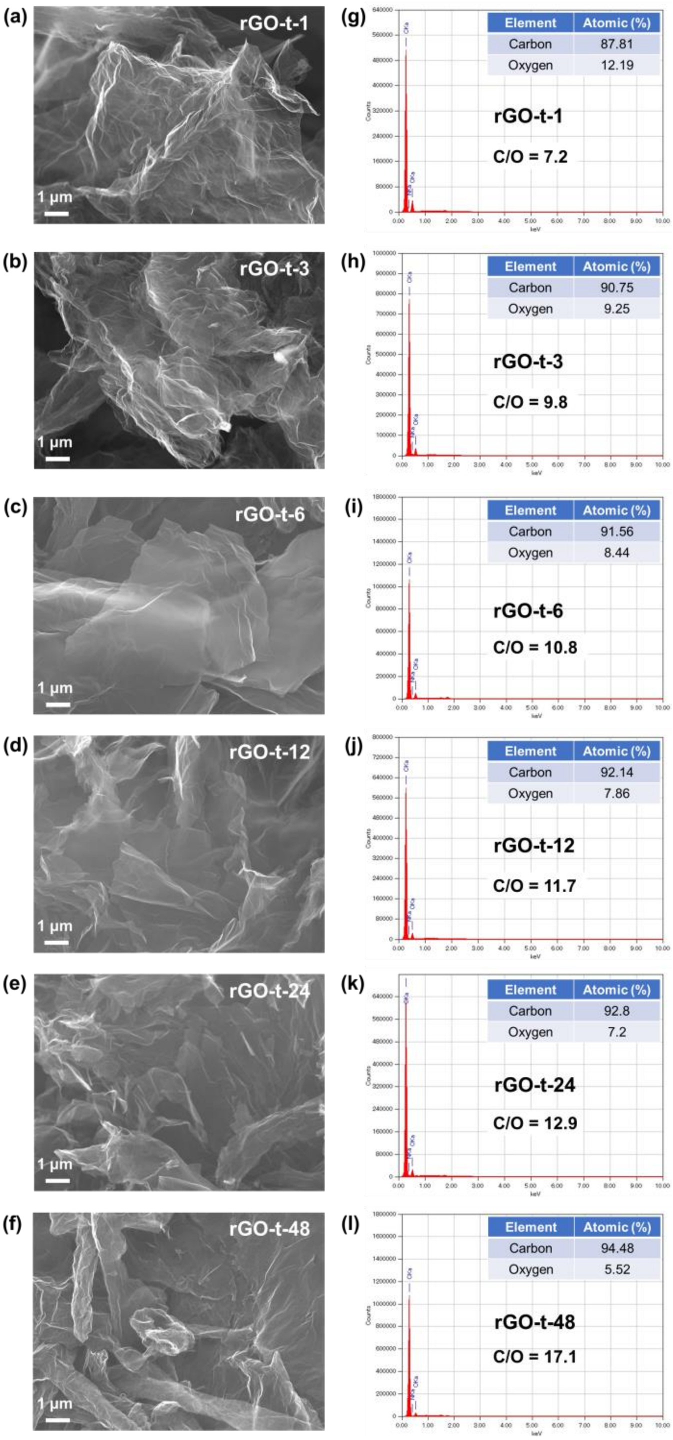

Fig. S4. (a-f) SEM images, (g-l) EDS measurements, and chemical compositions (inset) of rGOs.

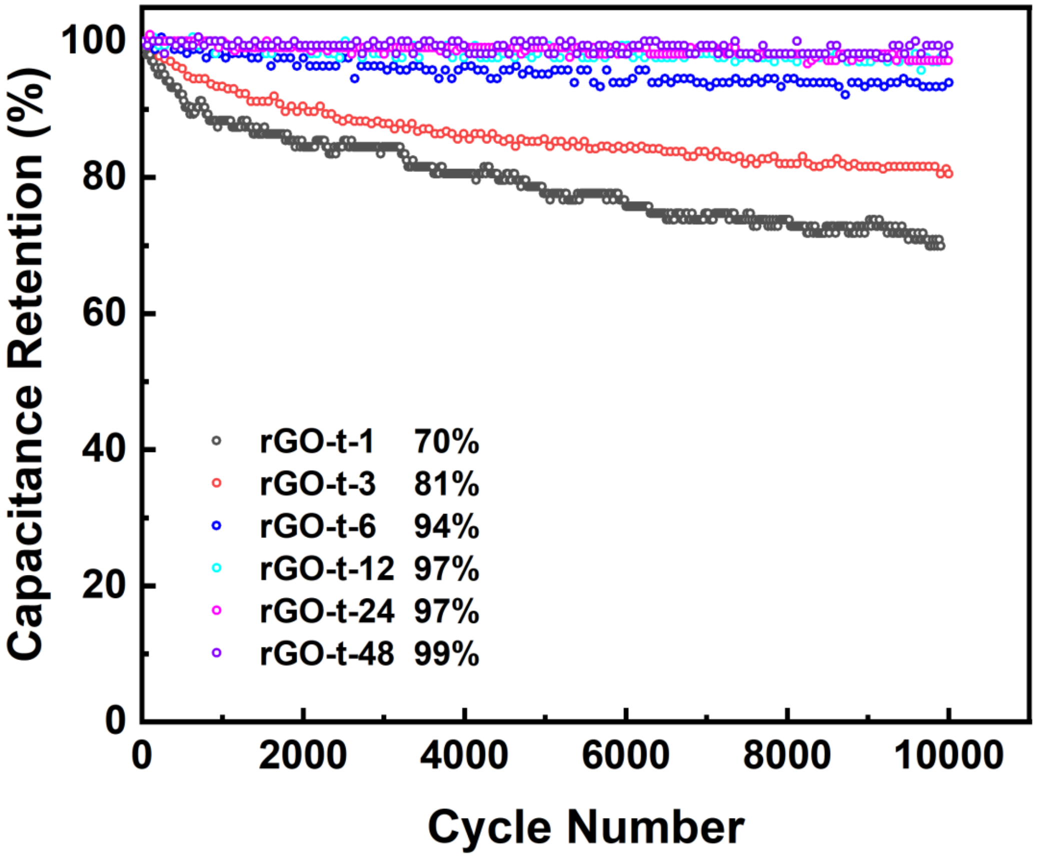

Fig. S5. Cyclic stability of supercapacitors with rGO electrodes.
